# Early Antibiotic Exposure and Bronchopulmonary Dysplasia in Very Preterm Infants at Low Risk of Early-Onset Sepsis

**DOI:** 10.1001/jamanetworkopen.2024.18831

**Published:** 2024-06-27

**Authors:** Wei Shi, Zheng Chen, Liping Shi, Siyuan Jiang, Jianguo Zhou, Xinyue Gu, Xiaoping Lei, Tiantian Xiao, Yanping Zhu, Aimin Qian, Wenhao Zhou, Shoo K. Lee, Lizhong Du, Jie Yang, Xiaolu Ma, Liyuan Hu

**Affiliations:** 1 Neonatal Intensive Care Unit, Children’s Hospital, Zhejiang University School of Medicine, Hangzhou, China; 2 National Clinical Research Center for Child Health, Hangzhou, China; 3 Department of Neonatology, Children’s Hospital of Fudan University, Shanghai, China; 4 National Health Commission Key Laboratory of Neonatal Diseases, Fudan University, Children’s Hospital of Fudan University, Shanghai, China; 5 Division of Neonatology, Department of Pediatrics, The Affiliated Hospital of Southwest Medical University, Luzhou, China; 6 Department of Neonatology, Chengdu Women’s and Children’s Central Hospital, School of Medicine, University of Electronic Science and Technology of China, Chengdu, China; 7 Department of Neonatology, The First Affiliated Hospital of Xinjiang Medical University, Urumqi, China; 8 Department of Neonatology, Children’s Hospital of Nanjing Medical University, Nanjing, China; 9 Maternal-Infant Care Research Center and Department of Pediatrics, Mount Sinai Hospital, Toronto, Ontario, Canada

## Abstract

**Importance:**

The overutilization of antibiotics in very preterm infants (VPIs) at low risk of early-onset sepsis (EOS) is associated with increased mortality and morbidities. Nevertheless, the association of early antibiotic exposure with bronchopulmonary dysplasia (BPD) remains equivocal.

**Objective:**

To evaluate the association of varying durations and types of early antibiotic exposure with the incidence of BPD in VPIs at low risk of EOS.

**Design, Setting, and Participants:**

This national multicenter cohort study utilized data from the Chinese Neonatal Network (CHNN) which prospectively collected data from January 1, 2019, to December 31, 2021. VPIs less than 32 weeks’ gestational age or with birth weight less than 1500 g at low risk of EOS, defined as those born via cesarean delivery, without labor or rupture of membranes, and no clinical evidence of chorioamnionitis, were included. Data analysis was conducted from October 2022 to December 2023.

**Exposure:**

Early antibiotic exposure was defined as the total number of calendar days antibiotics were administered within the first week of life, which were further categorized as no exposure, 1 to 4 days of exposure, and 5 to 7 days of exposure.

**Main Outcomes and Measures:**

The primary outcome was the composite of moderate to severe BPD or mortality at 36 weeks’ post menstrual age (PMA). Logistic regression was employed to assess factors associated with BPD or mortality using 2 different models.

**Results:**

Of the 27 176 VPIs included in the CHNN during the study period (14 874 male [54.7%] and 12 302 female [45.3%]), 6510 (23.9%; 3373 male [51.8%] and 3137 female [48.2.%]) were categorized as low risk for EOS. Among them, 1324 (20.3%) had no antibiotic exposure, 1134 (17.4%) received 1 to 4 days of antibiotics treatment, and 4052 (62.2%) received 5 to 7 days of antibiotics treatment. Of the 5186 VPIs who received antibiotics, 4098 (79.0%) received broad-spectrum antibiotics, 888 (17.1%) received narrow-spectrum antibiotics, and 200 (3.9%) received antifungals or other antibiotics. Prolonged exposure (5-7 days) was associated with increased likelihood of moderate to severe BPD or death (adjusted odds ratio [aOR], 1.23; 95% CI, 1.01-1.50). The use of broad-spectrum antibiotics (1-7 days) was also associated with a higher risk of moderate to severe BPD or death (aOR, 1.27; 95% CI, 1.04-1.55).

**Conclusions and Relevance:**

In this cohort study of VPIs at low risk for EOS, exposure to prolonged or broad-spectrum antibiotics was associated with increased risk of developing moderate to severe BPD or mortality. These findings suggest that VPIs exposed to prolonged or broad-spectrum antibiotics early in life should be monitored for adverse outcomes.

## Key Points

**Question:**

Is early antibiotic exposure associated with higher risk of bronchopulmonary dysplasia (BPD) in very preterm infants (VPIs) at low risk of early-onset sepsis (EOS)?

**Findings:**

In this cohort study that included 27 176 VPIs in China, prolonged early antibiotic exposure (5-7 days) was associated with increased likelihood of moderate to severe BPD or death among VPIs at low risk of EOS. The use of broad-spectrum antibiotics (1-7 days) was also associated with a higher risk of moderate to severe BPD or death.

**Meaning:**

The results suggest that careful monitoring is necessary for adverse outcomes of BPD or mortality among VPIs with low risk of EOS when given prolonged or broad-spectrum antibiotics treatment in early life.

## Teaser

This cohort study assesses whether various types of antibiotics and the length of exposure are associated with the incidence of bronchopulmonary dysplasia (BPD) among very preterm infants at low risk of early-onset sepsis (EOS) in China.

## Introduction

Early-onset sepsis (EOS) poses a substantial risk to very preterm infants, necessitating the prevalent administration of empirical antibiotics in the neonatal intensive care unit (NICU). Puopolo et al^1^ reported that more than one-third of the extremely preterm infants in their study were categorized as low risk for EOS, with an incidence of 0.5%. In these low-risk groups, prolonged empirical antibiotic treatment (≥5 days) was observed in approximately 35% of cases.^1^ Recent studies^1,2,3^ have suggested a potential association of prolonged antibiotic therapy with both increased mortality and a higher incidence of adverse neonatal outcomes, including bronchopulmonary dysplasia (BPD), within this vulnerable population. However, these studies often relegated BPD as a secondary outcome, lacking comprehensive consideration of relevant confounding factors. Moreover, the literature has reported inconsistent results in this domain.^4,5,6^ The reported association may be influenced by factors such as culture-proven sepsis bacteremia, illness severity, and early respiratory disease.

Moreover, the underlying mechanism of this association remains incompletely understood. One proposed explanation is that early antibiotic exposure may disrupt the neonatal intestinal microbiome, leading to dysregulation of innate immune mechanisms that protect against airway and systemic inflammation through the intestinal-pulmonary axis, ultimately heightening the risk of BPD.^7,8^ Comparing the potential harm caused by the overuse of broad-spectrum antibiotics on beneficial microbial communities,^9^ narrow-spectrum antibiotics may offer a more protective alternative by preserving the integrity of these microbial communities.^10^

To our knowledge, there is limited data on the impact of different classes of antibiotic exposure on the risk of BPD, particularly among infants at low risk of EOS. Our aim is to examine the association of the duration and types of early life antibiotic exposure with the incidence of mortality or BPD in very preterm infants at low risk of EOS.

## Methods

### Study Design, Setting, and Data Collection

This cohort study was approved by the Ethics Committee of the Children’s Hospital of Fudan University and was endorsed by all participating centers. The study was reported following the Strengthening the Reporting of Observational Studies in Epidemiology (STROBE) reporting guideline. This cohort utilized the database of the Chinese Neonatal Network (CHNN), which has prospectively collected data since its inception on January 1, 2019, through December 31, 2021. The follow-up time of the study was death or discharge from NICUs of CHNN hospitals. Initially, the CHNN comprised 57 NICUs, all of which contributed comprehensive data on infants who were very preterm or had very low birth weight.^11^ This number expanded to 70 NICUs in 2020 and further increased to 79 NICUs in 2021. All clinical data were collected from patient medical records according to the abstractors’ manuals by trained data abstractors.^12^ Given the utilization of deidentified patient data, a waiver of informed consent was granted at all sites.

### Participants

Infants born at less than 32 weeks’ gestational age (GA) or who had birth weights less than 1500 g and were considered to be at low risk of EOS were included. Infants categorized as low-risk exhibited the following characteristics: delivery via cesarean delivery, no rupture of membranes at the time of delivery, and absence of any clinical features of chorioamnionitis from the obstetric history.^1^ Furthermore, infants with major congenital anomalies, those who received inotropic therapy, died, or were discharged within 7 days after birth were excluded. Infants with culture-proven sepsis, *Ureaplasma* infection, necrotizing enterocolitis, or spontaneous intestinal perforation within the first 7 days after birth were also excluded.

### Exposures

Early antibiotic exposure was defined as the administration of antibiotic treatment within the initial 7 days postbirth. The duration of antibiotic exposure referred to the cumulative number of days within this 7-day period during which antibiotics were administered, irrespective of whether these days were sequential. A threshold of 4 calendar days was established to distinguish between short-term (1-4 days) and extended (5-7 days) antibiotic exposure during the neonate’s first week.

Broad-spectrum antibiotics referred to extended-spectrum penicillins with β-lactamase inhibitors, third-generation cephalosporins, fourth-generation cephalosporins, carbapenems, linezolid, and vancomycin. All other antibiotics, except for antifungal drugs and those with unknown classification, were regarded as narrow-spectrum antibiotics (eTable 1 in Supplement 1).

### Outcomes

Our primary outcome was a composite outcome of moderate to severe BPD or mortality at 36 weeks’ postmenstrual age (PMA). Moderate to severe BPD was defined as the necessity for supplemental oxygen for a minimum of 28 days, coupled with a continued requirement for oxygen or ventilatory support at 36 weeks’ PMA.^13^ For infants discharged or transferred before 36 weeks’ PMA, BPD was determined based on available data, or alternatively, their need for supplemental oxygen at the time of discharge or transfer. Secondary outcomes included moderate to severe BPD in survivors at 36 weeks’ PMA, mortality prior to 36 weeks’ PMA, mortality before CHNN hospital discharge, the need for home oxygen therapy, the total duration of mechanical ventilatory support (days), the cumulative number of days necessitating oxygen supplementation until discharge, and the length of CHNN hospital stay.

### Covariates

The study variables employed in this study were defined following the protocols established in the CHNN abstractor’s manual.^14^ GA was determined using the most reliable estimate available, based on prenatal ultrasonography, menstrual history, obstetric examination, or a combination of these methodologies. In cases where the obstetric estimate of GA deviated by more than 2 weeks from the postnatal neonatal assessment or when the obstetric estimate was not available, the Ballard Score was utilized to estimate the GA.^15^ Small for GA (SGA) was defined as birth weight less than the tenth percentile for GA and sex, according to the Chinese neonatal birth weight standards.^16^ *Ureaplasma* infection was characterized by a positive *Ureaplasma* culture or polymerase chain reaction test in tracheal aspirate samples, followed by treatment with macrolide antibiotics. Necrotizing enterocolitis was defined as stage II or above, in accordance with the Bell criteria.^17^ Patent ductus arteriosus (PDA) was identified through echocardiogram, confirming the presence of a ductus arteriosus, and necessitating either pharmacological intervention or surgical ligation.^18^ In our study, the term *mechanical ventilation support* encompassed both conventional mechanical ventilation and high-frequency oscillatory ventilation.

### Statistical Analysis

The study population was stratified into 3 distinct groups based on the duration of antibiotic administration (0 days, 1-4 days, or 5-7 days). Maternal and neonatal characteristics as well as antibiotic regimens were compared among the 3 groups. Frequencies (percentages) or medians (IQRs) were reported. Difference was assessed by Pearson χ^2^ or Kruskal-Wallis test for categorical variables and continuous variables, respectively.

Logistic regression was employed to assess the risk factors for BPD or mortality. The group without antibiotic exposure (0 days) was used as the reference when assessing the impact of short-term (1-4 days) and extended (5-7 days) antibiotic exposure during the neonate’s first week. Two regression models were developed to adjust for potential confounders that could affect the risk of BPD. Model 1 included adjustments for GA; birth weight; SGA; sex; multiple pregnancy; maternal age; gestational diabetes; gestational hypertension, preeclampsia, or eclampsia; antenatal corticosteroid use; and magnesium sulfate use. To account for the potential confounding effect of illness severity, our primary analysis (model 2) included additional adjustments for various factors, including Apgar score, intubation in the delivery room, treatment with surfactant or nitric oxide, PDA requiring pharmacological treatment, and mechanical ventilation treatment within 7 days after birth. Adjusted odds ratios (aORs) with 95% CIs were calculated. Generalized linear regression was utilized to analyze the continuous variables within the secondary outcome indicators associated with BPD, specifically when the duration of antibiotic exposure was treated as a continuous variable. Regression coefficients were expressed as β values with 95% CIs, reflecting the estimated mean differences for continuous outcomes. Generalized estimating equation models (model 3) were conducted to adjust for center cluster effect in addition to the confounders in model 2.

To evaluate the robustness of our findings, we conducted several sensitivity analyses. First, logistic regressions were applied to assess the primary outcomes based on different classification criteria for the duration of antibiotic exposure (criteria 1: 0 days, 1-3 days, or 4-7 days; criteria 2: 0 days, 1-4 days, or 5-7 days; criteria 3: 0 days, 1-5 days, or 6-7 days). Second, we employed propensity score matching to equalize the baseline characteristics among 3 groups (0 days, 1-4 days, or 5-7 days), with the objective of evaluating the consistency of the results in the propensity score-matched sample. Third, we focused on the association of initial antibiotic therapy with the risk of BPD, defining initial antibiotic therapy as starting within the first 3 days after birth, and conducted sensitivity analysis on the primary outcome using a propensity score model. Fourth, we conducted further analysis specifically focusing on centers with relatively stricter criteria for antibiotic therapy to account for the potential association of center-specific factors with the outcomes. The study included 14 centers, and the median proportion of prolonged antibiotic exposure (5-7 days) in infants was described. Finally, there were 728 participants (11.2%) with missing data for magnesium sulfate during delivery admission, while the proportion of missing data for other variables was less than 10% and were considered missing at random. In the analysis, missing data values were not imputed because of the low missing proportion. To validate the regression adjustment results, we conducted sensitivity analyses in a subsample with imputed missing data for magnesium sulfate during delivery admission, treating them as none due to their likely nonevent nature. Propensity score modeling was then used to assess the primary outcomes.

Additionally, the effect of different classes of antibiotics was assessed. The study population was divided into 2 groups by different classes of antibiotics (broad-spectrum or narrow-spectrum antibiotics) within the first 7 days of life. Infants who received a mix of broad and narrow-spectrum antibiotics within the first week were included in the broad-spectrum antibiotic group. Regression models were employed. Additionally, a stratified analysis was conducted to explore variations in the effects of broad-spectrum or narrow-spectrum antibiotics based on the duration of antibiotic exposure (1-4 days or 5-7 days).

All analyses were conducted using SAS software version 9.4 (SAS Institute), with a 2-sided significance level set at *P* < .05. Data analysis was conducted from October 2022 to December 2023.

## Results

### Characteristics of Study Population

This study included a cohort of 27 176 infants (14 874 males [54.7%] and 12 302 females [45.3%]) with birth weight less than 1500 g or GA less than 32 weeks who were admitted within the first 24 hours of life to CHNN centers from January 1, 2019, to December 31, 2021. After excluding those at risk for or with early sepsis, with major congenital anomalies, and other additional exclusion criteria, 6510 infants (23.9%) were categorized as low risk for EOS (3373 male [51.8%] and 3137 female [48.2.%]) and were included (Figure). Of these, 1324 (20.3%) received no antibiotics, 1134 (17.4%) were treated with antibiotics for 1 to 4 days, and 4052 (62.2%) received antibiotics for a duration of 5 to 7 days (Figure).

**Figure.**
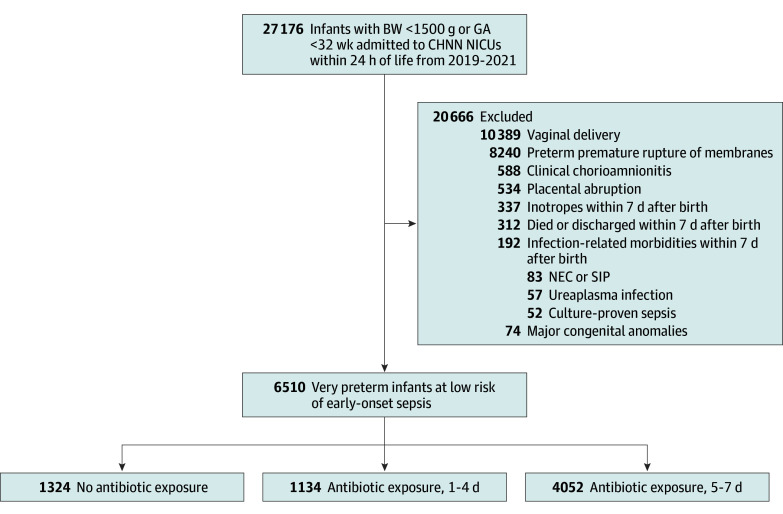
Study Cohort Flow Diagram BW indicates birth weight; CHNN, Chinese Neonatal Network; GA, gestational age; NEC, necrotizing enterocolitis; NICU, neonatal intensive care unit; SIP, spontaneous intestinal perforation.

The maternal and infant characteristics of the 3 groups are summarized in Table 1. The prolonged antibiotic treatment group (5-7 days) included neonates with lower GA and birth weight, a decreased incidence of SGA, and lower Apgar scores. Additionally, these infants were more likely to require intubation at birth, mechanical ventilation during the first week of life, treatments involving surfactant and nitric oxide, as well as medical treatment for PDA. Moreover, the mothers of these infants tended to be older and less likely to have received corticosteroids or magnesium sulfate prior to delivery.

**Table 1.** Baseline Characteristics of Participants

Variables	Early antibiotic exposure, No./No. (%) (N = 6510)			*P* value
	None (n = 1324)	1-4 d (n = 1134)	5-7 d (n = 4052)	
Maternal characteristics				
Maternal age ≥35 y	317/1309 (24.2)	246/1125 (21.9)	1053/4034 (26.1)	.01
Multiple pregnancy	417/1324 (31.5)	355/1134 (31.3)	1070/4052 (26.4)	<.001
Gestational diabetes	242/1320 (18.3)	211/1127 (18.7)	763/4023 (19.0)	.88
Antenatal corticosteroids	1059/1279 (82.8)	910/1088(83.6)	3051/3810 (80.1)	.01
Gestational hypertension, preeclampsia, or eclampsia	707/1319 (53.6)	511/1128 (45.3)	1916/4029 (47.6)	<.001
Magnesium sulfate during delivery admission	760/1232 (61.7)	625/1020 (61.3)	2011/3530 (57.0)	.003
Neonatal characteristics				
Birth weight, g				
<1000	100/1324 (7.6)	119/1134 (10.5)	580/4052 (14.3)	<.001
≥1000	1224/1324 (92.4)	1015/1134 (89.5)	3472/4052 (85.7)	
Gestational age, wk				
<28	33/1324 (2.5)	56/1134 (4.9)	222/4052 (5.5)	<.001
≥28	1291/1324 (97.5)	1078/1134 (95.1)	3830/4052 (94.5)	
Small for gestational age	538/1324 (40.6)	337/1134 (29.7)	1122/4052 (27.7)	<.001
Sex				
Male	607/1324 (45.8)	554/1134 (48.9)	2212/4052 (54.6)	<.001
Female	717/1324 (54.2)	580/1134 (51.1)	180/4052 (45.4)	
Apgar score <7 at 5 min	15/1264 (1.2)	47/1096 (4.3)	275/3940 (7.0)	<.001
Intubation in delivery room	135/1309 (10.3)	158/1115 (14.2)	1021/3974 (25.7)	<.001
Mechanical ventilation treatment in 7 d after birth	154/1324 (11.6)	216/1134 (19.1)	1540/4052 (36.0)	<.001
Surfactant use	355/1324 (26.8)	520/1134 (45.9)	2276/4052 (56.2)	<.001
Nitric oxide use	1/1324 (0.1)	4/1134 (0.4)	26/4052 (0.6)	.03
Patent ductus arteriosus medication	146/1319 (11.1)	138/1132 (12.2)	567/4045 (14.0)	.01
Patent ductus arteriosus ligation	3/1324 (0.2)	5/1134 (0.4)	9/4052 (0.2)	.43

### Characteristics of Antibiotic Regimen

The median (IQR) proportion of infants receiving prolonged antibiotic treatment (5-7 days) within the first week of life was 90.9% (72.4%-98.0%) (eFigure 1 in Supplement 1). Predominant use of broad-spectrum antibiotics over narrow-spectrum antibiotics was observed consistently among infants with varying duration of antibiotics (eFigure 2 in Supplement 1), with third-generation cephalosporins and extended-spectrum penicillins with β-lactamase inhibitors being the most frequently used classes.

### Association of Early Antibiotic Exposure With BPD

The incidence of the primary outcome is reported in Table 2. After adjusting for infant demographic characteristics and maternal characteristics (model 1), within 7 days of life, the prolonged antibiotic exposure group (5-7 days) showed a significantly higher risk of moderate to severe BPD or death compared with those with no antibiotic exposure (aOR, 1.83; 95% CI, 1.53-2.19) or short-term exposure (1-4 days; aOR, 1.74; 95% CI, 1.44-2.09). This association remained significant after incorporating additional confounding variables in model 2 when comparing early antibiotic exposure of 5 to 7 days vs none (aOR ,1.23; 95% CI, 1.01-1.50) and 5 to 7 days vs 1 to 4 days (aOR, 1.40; 95% CI, 1.15-1.71). After adjusting for center cluster effect, an increased risk of moderate to severe BPD or death remained (eTable 2 in Supplement 1).

**Table 2.** Durations of Early Antibiotic Exposure and Neonatal Outcomes Among Infants With Low Risk of Early-Onset Sepsis^a^

Outcomes	Early antibiotic exposure, No./No. (%) (N = 6510)			Model 1, aOR (95% CI)^b^			Model 2, aOR (95% CI)^c^		
	None (n = 1324)	1-4 d (n = 1134)	5-7 d (n = 4052)	1-4 d vs none	5-7 d vs none	5-7 d vs 1-4 d	1-4 d vs none	5-7 d vs none	5-7 d vs 1-4 d
Moderate to severe BPD or death	208/1324 (15.7)	211/1134 (18.6)	1165/4052 (28.8)	1.05 (0.83 to 1.33)	1.83 (1.53 to 2.19)	1.74 (1.44 to 2.09)	0.88 (0.68 to 1.13)	1.23 (1.01 to 1.50)	1.40 (1.15 to 1.71)
Moderate to severe BPD in survivors at 36 wk PMA	192/1276 (15.1)	188/1111 (16.9)	1034/3926 (26.3)	0.98 (0.77 to 1.25)	1.72 (1.43 to 2.07)	1.75 (1.44 to 2.13)	0.85 (0.65 to 1.10)	1.19 (0.97 to 1.46)	1.40 (1.14 to 1.73)
Death prior to 36 wk PMA	13/1324 (1.0)	23/1134 (2.0)	126/4052 (3.1)	2.04 (0.97 to 4.28)	2.51 (1.33 to 4.74)	1.23 (0.76 to 2.00)	1.61 (0.73 to 3.56)	1.73 (0.88 to 3.43)	1.08 (0.64 to 1.82)
Death before hospital discharge	19/1324 (1.4)	25/1134 (2.2)	147/4052 (3.6)	1.60 (0.82 to 3.11)	2.28 (1.31 to 3.94)	1.43 (0.90 to 2.27)	1.28 (0.63 to 2.60)	1.59 (0.89 to 2.86)	1.24 (0.75 to 2.06)
Home oxygen therapy	32/1324 (2.4)	44/1079 (4.1)	209/3775 (5.4)	1.71 (1.01 to 2.90)^d^	2.36 (1.52 to 3.65)^d^	1.38 (0.95 to 2.00)^d^	1.47 (0.84 to 2.58)^d^	1.84 (1.15 to 2.93)^d^	1.25 (0.83 to 1.88)^d^
Mechanical ventilation support, median (IQR), d	4 (2 to 8)	3 (2 to 7)	4 (2 to 8)	−0.83 (−2.74 to 1.09)^d^	−2.14 (−3.64 to −0.64)^d^	−1.31 (−2.73 to 0.10)^d^	−0.02 (−2.00 to 1.95)^d^	−1.53 (−3.12 to 0.07)^d^	−1.51 (−2.94 to −0.07)^d^
Oxygen supplementation, median (IQR), d	15 (8 to 29)	16 (7 to 31)	22 (10 to 38)	−0.47 (−2.07 to 1.13)^d^	3.54 (2.27 to 4.81)^d^	4.01 (2.70 to 5.31)^d^	−1.58 (−3.10 to −0.06)^d^	0.11 (−1.14 to 1.35)^d^	1.69 (0.44 to 2.93)^d^
Length of hospital stay, median (IQR), d	37 (29 to 48)	41 (32 to 53)	46 (36 to 59)	1.84 (0.23 to 3.45)^d^	4.79 (3.52 to 6.07)^d^	2.95 (1.60 to 4.30)^d^	1.04 (−0.58 to 2.66)^d^	2.73 (1.41 to 4.05)^d^	1.69 (0.33 to 3.05)^d^

Abbreviations: aOR, adjusted odds ratio; BPD, bronchopulmonary dysplasia; PMA, postmenstrual age.

^a^ Logistic regression models were used to analyze the categorical variables, while generalized linear regression models were used to analyze the continuous variables.

^b^ Adjusted for gestational age, birthweight, small for gestational age, sex, multiple pregnancy, maternal age, gestational diabetes, gestational hypertension, preeclampsia or eclampsia, antenatal corticosteroids use, and magnesium sulfate use.

^c^ Adjusted for same variables as model 1 plus Apgar score, intubation in delivery room, treatment with surfactant, nitric oxide, patent ductus arteriosus requiring pharmacological treatment, and mechanical ventilation treatment in 7 days after birth.

^d^ Results presented as β (95% CI).

In survivors at 36 weeks’ PMA, prolonged antibiotic exposure was consistently associated with a higher risk of moderate to severe BPD in both models (aOR for model 1, 1.72; 95% CI, 1.43-2.07; aOR for model 2, 1.19; 95% CI, 0.97-1.46). However, neither prolonged nor short antibiotic exposure was associated with an increased risk of death prior to 36 weeks’ PMA or before discharge (Table 2). Compared with no antibiotic therapy, treatment with antibiotics for 5 to 7 days (but not 1 to 4 days) was associated with longer hospital stays (β = 2.73; 95% CI, 1.41-4.05) and a higher likelihood of infants requiring home oxygen therapy (β = 1.84; 95% CI, 1.15-2.93). An analysis of antibiotic exposure as a continuous variable (Table 3) revealed that each additional day of antibiotic use within the first week was associated with 10% increased odds of the primary composite outcome and 10% higher odds of moderate to severe BPD in survivors to 36 weeks in model 2.

**Table 3.** Association of Each Additional Day of Early Antibiotic Exposure With Neonatal Outcomes Among Infants With Low Risk of Early-Onset Sepsis

Outcomes^a^	Unadjusted OR (95% CI)	aOR (95% CI)	
		Model 1^b^	Model 2^c^
Moderate to severe BPD or death	1.17 (1.12-1.22)	1.16 (1.10-1.21)	1.10 (1.04-1.16)
Moderate to severe BPD in survivors at 36 wk PMA	1.17 (1.12-1.23)	1.16 (1.10-1.22)	1.10 (1.04-1.16)
Death prior to 36 wk PMA	1.11 (0.99-1.24)	1.04 (0.92-1.18)	1.00 (0.87-1.14)
Death before hospital discharge	1.12 (1.01-1.25)	1.08 (0.96-1.21)	1.04 (0.91-1.18)

Abbreviations: aOR, adjusted odds ratio; BPD, bronchopulmonary dysplasia; OR, odds ratio; PMA, postmenstrual age.

^a^ Logistic regression models were used to analyze the categorical variables.

^b^ Adjusted for gestational age, birthweight, small for gestational age, sex, multiple pregnancy, maternal age, gestational diabetes, gestational hypertension, preeclampsia or eclampsia, antenatal corticosteroids use, and magnesium sulfate use.

^c^ Adjusted for same variables as model 1 plus Apgar score, intubation in delivery room, treatment with surfactant, nitric oxide, patent ductus arteriosus requiring pharmacological treatment, and mechanical ventilation treatment in 7 days after birth.

In all sensitivity analyses, including different cutoffs (3, 4, and 5 calendar days) to define short and prolonged antibiotic exposure, propensity score matching, limiting to infants with early antibiotic exposure starting within the first 3 days after birth, and imputation of missing data for magnesium sulfate during delivery admission, the results remained consistent for the primary composite outcome when comparing infants receiving prolonged antibiotic treatment with infants not receiving antibiotics (eFigure 3 and eTables 3-8 in Supplement 1). To examine the association of the center with outcomes, we ranked the centers by proportion of antibiotics used in first 7 days of life and assessed the top 14 centers that implemented relatively stricter criteria for antibiotic therapy. The characteristics of the patients in these 14 centers are presented in eTable 9 in Supplement 1. Within this cluster of centers, the median (IQR) proportion of infants with prolonged antibiotic exposure (5-7 days) was 41.2% (23.3%-58.3%). Similar results were identified when analyzing the primary outcome in this population, as indicated in eTable 10 in Supplement 1.

### Different Associations of Broad-Spectrum and Narrow-Spectrum Antibiotics With BPD

Of the 5186 infants who received antibiotics in the study cohort, 888 (17.1%) received narrow-spectrum antibiotics, 4098 (79.0%) received broad-spectrum antibiotics, and 200 (3.9%) received antifungals or other antibiotics within the first week of life (Table 4). Compared with infants without early antibiotics exposure, infants with broad-spectrum antibiotics exposure (1-7 days) had an increased risk of moderate to severe BPD or death at 36 weeks’ PMA (1200 of 4098 infants [29.3%] vs 208 of 1324 infants [15.7%]; aOR 1.27; 95% CI, 1.04-1.55; model 2). In addition, broad-spectrum antibiotic exposure in the early period was also associated with an increased risk of moderate to severe BPD in survivors at 36 weeks’ PMA. However, these associations were not observed when infants only received narrow-spectrum antibiotics within 7 days of life.

**Table 4.** Different Types of Early Antibiotic Exposure and Neonatal Outcomes Among Infants With Low Risk of Early-Onset Sepsis

Outcomes^c^	Early antibiotic exposure, No./No. (%) (N = 6510)			Model 1, aOR (95% CI)^a^		Model 2, aOR (95% CI)^b^	
	None (n = 1324)	Narrow-spectrum (n = 888)	Broad-spectrum (n = 4098)	Narrow-spectrum vs none	Broad-spectrum vs none	Narrow-spectrum vs none	Broad-spectrum vs none
Moderate to severe BPD or death	208/1324 (15.7)	168/888 (18.9)	1200/4098 (29.3)	1.19 (0.93-1.51)	1.86 (1.55-2.23)	0.98 (0.75-1.27)	1.27 (1.04-1.55)
Moderate to severe BPD in survivors at 36 wk PMA	192/1276 (15.1)	149/870 (17.1)	1067/3969 (26.9)	1.12 (0.87-1.44)	1.75 (1.46-2.11)	0.94 (0.71-1.22)	1.24 (1.01-1.52)
Death prior to 3 wk PMA	13/1324 (1.0)	18/888 (2.0)	129/4098 (3.2)	2.02 (0.93-4.36)	2.54 (1.35-4.78)	1.61 (0.72-3.61)	1.75 (0.88-3.45)
Death before hospital discharge	19/1324 (1.4)	20/888 (2.3)	150/4098 (3.7)	1.61 (0.80-3.24)	2.29 (1.32-3.97)	1.33 (0.65-2.75)	1.60 (0.89-2.87)

Abbreviations: aOR, adjusted odds ratio; BPD, bronchopulmonary dysplasia; PMA, postmenstrual age.

^a^ Adjusted for gestational age, birthweight, small for gestational age, sex, multiple pregnancy, maternal age, gestational diabetes, gestational hypertension, preeclampsia or eclampsia, antenatal corticosteroids use, and magnesium sulfate use.

^b^ Adjusted for same variables as model 1 plus Apgar score, intubation in delivery room, treatment with surfactant, nitric oxide, patent ductus arteriosus requiring pharmacological treatment, and mechanical ventilation treatment in 7 days after birth.

^c^ Logistic regression models were used to analyze the categorical variables.

Subgroup analyses further showed that prolonged exposure (5-7 days) to broad-spectrum antibiotics was associated with increased adjusted odds (model 2) of the primary composite outcome (aOR, 1.33; 95% CI, 1.08-1.63) and moderate to severe BPD in survivors at 36 weeks’ PMA (aOR, 1.29; 95% CI, 1.04-1.59) (eFigure 4 in Supplement 1). These associations were not present when examining infants receiving short-term broad-spectrum antibiotic therapy. Additionally, in the subgroup analyses for infants with narrow-spectrum antibiotic exposure, there was no association of early antibiotic exposure (1-4 days or 5-7 days) with moderate to severe BPD.

## Discussion

In this cohort study, 62.9% of the very preterm infants at low risk of EOS were exposed to prolonged antibiotic treatment (5-7 days) within their first week of life. Such prolonged antibiotic exposure was associated with a higher risk of the composite outcome of moderate to severe BPD or death at 36 weeks’ PMA. This risk was more pronounced with the use of broad-spectrum antibiotics than with narrow-spectrum antibiotics. Due to immaturity of immune systems of very preterm infants and their reliance on invasive life support, EOS is significantly more prevalent in this group compared with full-term infants.^19^ Empirical antibiotic therapy is often initiated as a precaution against EOS.^20,21^ In a large multicenter Chinese cohort study of infants less than 34^+0^ weeks’ GA,^22^ 85% received early antibiotics, while two-thirds of the infants received prolonged antibiotics therapy (>5 days) within the first week after birth. Meanwhile, among those preterm infants at lower risk for EOS, approximately one-third of them received prolonged antibiotics in their early lives in developed countries.^1,2,3^ According to American Academy of Pediatrics guidelines,^19^ antibiotics should be discontinued by 36 to 48 hours if blood cultures are sterile in infants at low risk of EOS. Interestingly, our study observed an even higher proportion of low-risk infants (62.9%) receiving prolonged antibiotics, indicating a possible overuse in this population.

The potential adverse consequences of prolonged early antibiotic exposure are increasingly recognized in recent years.^23^ Numerous cohort studies^7,21,23,24,25^ have reported an association of prolonged antibiotic therapy with increased neonatal mortality and morbidity in very preterm infants. Our findings were consistent with those of previous studies involving infants at low risk, although the definition of prolonged antibiotic exposure was slightly different. For example, in a study encompassing 29 tertiary NICUs in the Canadian Neonatal Network,^3^ 31% of infants with very low birth weight at low risk of EOS received prolonged antibiotics (4-7 days) in the first 7 days after birth, while in a US cohort of infants with extremely low birth weight,^1^ prolonged early antibiotic therapy was defined as the administration of antibiotics for 5 days or more. The increased risk of BPD was reported in both of these studies.^1,3^ Furthermore, our findings of elevated risk associated with each additional day of antibiotic therapy was consistent with the findings from the study carried out by Cantey et al.^5^ Our research goes further by highlighting the potential harms with exposure to prolonged empirical broad-spectrum antibiotics without sound indications.

We also acknowledged that the association of BPD with early antibiotic exposure among very preterm infants has been challenged by some other studies. Drawing from the Optum Neonatal Database in a cohort study involving 4950 VPIs,^4^ it was suggested that early antibiotic exposure lacked an independent association with an elevated risk of BPD or mortality among very preterm infants without culture-confirmed sepsis. The potential association of early antibiotic exposure with BPD could be confounded by severity of illness and early respiratory disease.^4^ To address these concerns, this study only enrolled very preterm infants at low risk of EOS and excluded infants with infection-related morbidities or circulatory instability requiring inotropes within the first 7 days of life. Additionally, 2 models were established to thoroughly adjust for confounding variables. These measures helped to eliminate interference by illness severity and severe cardiovascular diseases. In addition, the outcomes could be confounded by the effect of centers because of the large variation in antibiotic strategies among them.^26^ We conducted a sensitivity analysis specifically focusing on centers with relatively strict criteria for antibiotic therapy, which was similar to those in developed countries.^3^ The results showed that the odds of moderate to severe BPD or death were even higher among infants with prolonged antibiotic exposure. The association enhancement may be attributed to the composition of the enrolled population, characterized by lower GA and birth weight, increased reliance on respiratory support, and elevated BPD risk across the 14 centers. Furthermore, it is noteworthy that the standard regimen for EOS in many centers worldwide typically involves ampicillin plus gentamicin. However, in China, the use of aminoglycosides in preterm infants has been prohibited. This differing antibiotic strategy could potentially contribute to variations in outcomes.

Our study highlights differences in empirical antibiotic choices for EOS between developed countries and our data. While ampicillin and gentamicin are commonly used in developed countries,^19,27,28^ our study observed a predominant use of broad-spectrum antibiotics. Previous research has shown that early exposure to third-generation cephalosporins is associated with an increased risk of death compared with ampicillin and gentamicin.^29^ Our study revealed that very preterm infants exposed to broad-spectrum antibiotics, rather than narrow-spectrum antibiotics, in their first week of life exhibited a heightened risk of developing moderate to severe BPD or mortality. Research has shown that early antibiotic exposure can disrupt the preterm microbiome, thereby precipitating adverse outcomes like BPD by influencing systemic inflammation via the gut-lung-brain axis.^7,24,30,31,32^ Additionally, prolonged use of broad-spectrum antibiotics is associated with an increased risk of colonization by antibiotic resistant organisms such as cephalosporin-resistant gram-negative bacteria, vancomycin-resistant *Enterococcus*, and carbapenem-resistant organisms.^33,34^ These findings suggest that the choice of antibiotics in the early life of very preterm infants may have implications for their risk of developing BPD or experiencing adverse outcomes. Clinicians should consider the potential impact of broad-spectrum antibiotics and be mindful of the associated risks, including the disruption of the microbiome and the potential for colonization with multi–drug resistant organisms.

### Strengths and Limitations

To our knowledge, this study is the first nationwide Chinese cohort study to explore the association of early antibiotic exposure with BPD in very preterm infants at low risk of EOS and provides invaluable insights to antibiotic stewardship. However, it is important to acknowledge the limitations of our study. First, being an observational study, it cannot establish causality between early antibiotic exposure and BPD. Second, our study excluded infants with severe illnesses, including those who underwent inotropic therapy, experienced mortality, or were discharged within 7 days after birth. In our model, mechanical ventilation treatment within the first 7 days after birth was used as an indicator of the severity of early respiratory disease. However, confounding still exists. Our data could not identify cases with a potential infection (negative bacteriology but positive inflammatory syndrome) or maternal colonization with group B *Streptococcus*, both of which might require extended antibiotic treatment. This introduces a bias because perinatal inflammation is acknowledged as a factor associated with increased risk for BPD. Third, BPD is multifactorial, and we were not able to understand the impact of other events like ventilator associated pneumonia, fluid overload, and exposure to hemodynamically significant PDA on the development of BPD. Further study is needed to understand whether reducing early antibiotic exposure and other simultaneous interventions can substantially improve the respiratory outcomes.

## Conclusions

Our findings highlight a concerning overuse of early antibiotics among very preterm infants at low risk for EOS in China. Prolonged treatment with broad-spectrum antibiotics in these infants is associated with a heightened risk of developing moderate to severe BPD or mortality. This finding underscores the importance of cautious antibiotic use, especially in the early life stages of vulnerable populations.

## Notes

Supplement 1. **eTable 1.** Antibiotic Characteristics **eFigure 1.** Variation in the Proportion of Infants With Prolonged Antibiotics Exposure (5-7 days) by CHNN Centers **eFigure 2.** Characteristics of Antibiotic Regimen **eTable 2.** Sensitivity Analysis After the Center Effect Was Adjusted **eFigure 3.** Sensitivity Analysis According to the Different Classification Criteria **eTable 3.** Baseline Characteristics of Different Durations of Early Antibiotic Exposure Groups in the Propensity Score Matched Sample **eTable 4.** Sensitivity Analysis of Different Durations of Early Antibiotic Exposure in the Propensity Score Matched Sample **eTable 5.** Baseline Characteristics of Different Durations of Initial Antibiotic Therapy Groups (Starting <72 Hours of Age) in the Propensity Score Matched Sample **eTable 6.** Sensitivity Analysis of Different Durations of Initial Antibiotic Therapy (Starting <72 Hours of Age) in the Propensity Score Matched Sample **eTable 7.** Baseline Characteristics of Different Durations of Early Antibiotic Exposure in the Propensity Score Matched Sample With Imputation of Missing Data for Magnesium Sulfate During Delivery Admission **eTable 8.** Sensitivity Analysis of Different Durations of Early Antibiotic Exposure in the Propensity Score Matched Sample With Imputation of Missing Data for Magnesium Sulfate During Delivery Admission **eTable 9.** Baseline Characteristics of Participants in the Top 14 Centers That Implemented Relatively Stricter Criteria for Antibiotic Therapy **eTable 10.** Sensitivity Analysis According to the Proportion of Infants With Prolonged Antibiotic Exposure **eFigure 4.** Stratified Analysis for Effects of Broad-Spectrum or Narrow-Spectrum Antibiotics Varied by the Duration of Antibiotics Exposure

Supplement 2.
Nonauthor Collaborators

Supplement 3.
Data Sharing Statement
