# Supplementary material for: Early Antibiotic Exposure and Bronchopulmonary Dysplasia in Very Preterm Infants at Low Risk of Early-Onset Sepsis
Source: JAMA Netw Open. 2024 Jun 27;7(6):e2418831. doi: 10.1001/jamanetworkopen.2024.18831 (PMC11211957; doi:10.1001/jamanetworkopen.2024.18831)
Supplement: Supplement 1. — eTable 1. Antibiotic Characteristics eFigure 1. Variation in the Proportion of Infants With Prolonged Antibiotics Exposure (5-7 days) by CHNN Centers eFigure 2. Characteristics of Antibiotic Regimen eTable 2. Sensitivity Analysis After the Center Effect Was Adjusted eFigure 3. Sensitivity Analysis According to the Different Classification Criteria eTable 3. Baseline Characteristics of Different Durations of Early Antibiotic Exposure Groups in the Propensity Score Matched Sample eTable 4. Sensitivity Analysis of Different Durations of Early Antibiotic Exposure in the Propensity Score Matched Sample eTable 5. Baseline Characteristics of Different Durations of Initial Antibiotic Therapy Groups (Starting <72 Hours of Age) in the Propensity Score Matched Sample eTable 6. Sensitivity Analysis of Different Durations of Initial Antibiotic Therapy (Starting <72 Hours of Age) in the Propensity Score Matched Sample eTable 7. Baseline Characteristics of Different Durations of Early Antibiotic Exposure in the Propensity Score Matched Sample With Imputation of Missing Data for Magnesium Sulfate During Delivery Admission eTable 8. Sensitivity Analysis of Different Durations of Early Antibiotic Exposure in the Propensity Score Matched Sample With Imputation of Missing Data for Magnesium Sulfate During Delivery Admission eTable 9. Baseline Characteristics of Participants in the Top 14 Centers That Implemented Relatively Stricter Criteria for Antibiotic Therapy eTable 10. Sensitivity Analysis According to the Proportion of Infants With Prolonged Antibiotic Exposure eFigure 4. Stratified Analysis for Effects of Broad-Spectrum or Narrow-Spectrum Antibiotics Varied by the Duration of Antibiotics Exposure [file jamanetwopen-e2418831-s001.pdf]

Supplemental Online Content

Shi W, Chen Z, Shi L, et al. Early antibiotic exposure and bronchopulmonary dysplasia in very preterm infants at low risk of early-onset sepsis. *JAMA Netw. Open*. 2024;7(6):e2418831. doi:10.1001/jamanetworkopen.2024.18831

eTable 1. Antibiotic Characteristics

eFigure 1. Variation in the Proportion of Infants With Prolonged Antibiotics Exposure (5 – 7 days) by CHNN Centers

eFigure 2. Characteristics of Antibiotic Regimen

eTable 2. Sensitivity Analysis After the Center Effect Was Adjusted

eFigure 3. Sensitivity Analysis According to the Different Classification Criteria

eTable 4. Baseline Characteristics of Different Durations of Early Antibiotic Exposure Groups in the Propensity Score Matched Sample

eTable 4. Sensitivity Analysis of Different Durations of Early Antibiotic Exposure in the Propensity Score Matched Sample

eTable 5. Baseline Characteristics of Different Durations of Initial Antibiotic Therapy Groups (Starting <72 Hours of Age) in the Propensity Score Matched Sample

eTable 6. Sensitivity Analysis of Different Durations of Initial Antibiotic Therapy (Starting <72 Hours of Age) in the Propensity Score Matched Sample

eTable 7. Baseline Characteristics of Different Durations of Early Antibiotic Exposure in the Propensity Score Matched Sample With Imputation of Missing Data for Magnesium Sulfate During Delivery Admission

eTable 8. Sensitivity Analysis of Different Durations of Early Antibiotic Exposure in the Propensity Score Matched Sample With Imputation of Missing Data for Magnesium Sulfate During Delivery Admission

eTable 9. Baseline Characteristics of Participants in the Top 14 Centers That Implemented Relatively Stricter Criteria for Antibiotic Therapy

eTable 10. Sensitivity Analysis According to the Proportion of Infants With Prolonged Antibiotic Exposure

eFigure 4. Stratified Analysis for Effects of Broad-Spectrum or Narrow-Spectrum Antibiotics Varied by the Duration of Antibiotics Exposure

This supplemental material has been provided by the authors to give readers additional information about their work.

© 2024 Shi W et al. *JAMA Network Open.*

eTable 1. Antibiotic Characteristics

Antibiotics characteristics	Early Antibiotic Exposure	
	1-4 Days (N=1134)	5-7 Days (N=4052)
**Broad-spectrum antibiotics, n (%)**	698 (61.6)	3400 (83.9)
Extended Spectrum Penicillin + β-	311 (27.4)	1358 (33.5)
Third-generation cephalosporins	411 (36.2)	2293 (56.6)
Fourth-generation cephalosporins	14 (1.2)	28 (0.7)
Carbapenems	31 (2.7)	478 (11.8)
Linezolid	0 (0.0)	4 (0.1)
Vancomycin	10 (0.9)	64 (1.6)
**Narrow-spectrum antibiotic, n (%)**	353 (31.1)	535 (13.2)
Penicillin/ Penicillinase-Resistant Penicillin	548 (48.3)	1856 (45.8)
First-generation cephalosporins	12 (1.1)	11 (0.3)
Second-generation cephalosporins	45 (4.0)	209 (5.2)
**Others, n (%)**	83 (7.3)	117 (2.9)

eFigure 1. Variation in the Proportion of Infants with Prolonged Antibiotics Exposure (5-7 days) by CHNN Centers

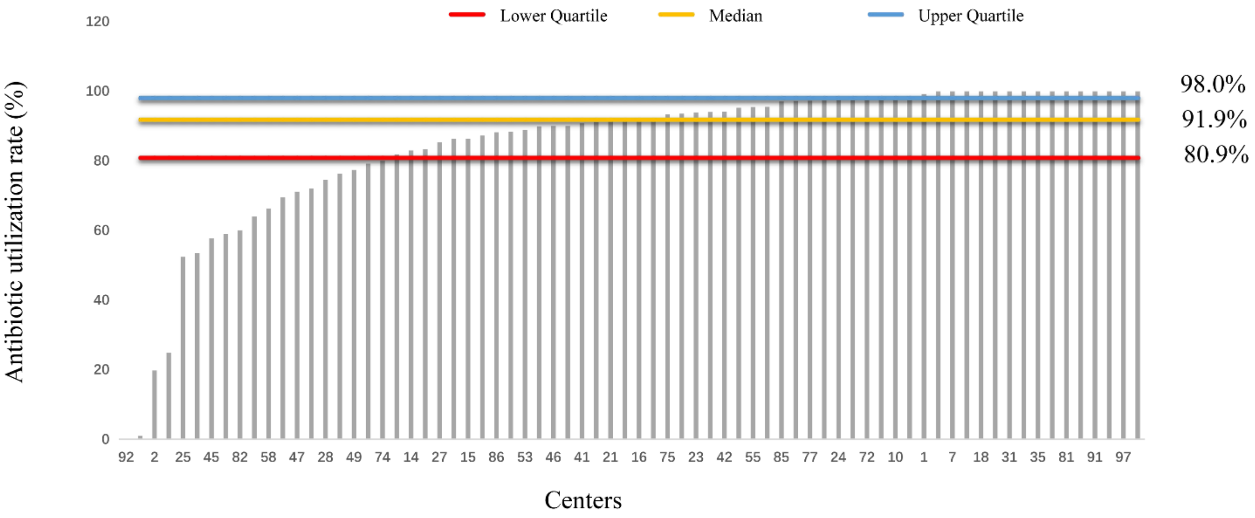

© 2024 Shi W et al. *JAMA Network Open.*

eFigure 2. Characteristics of Antibiotic Regimen

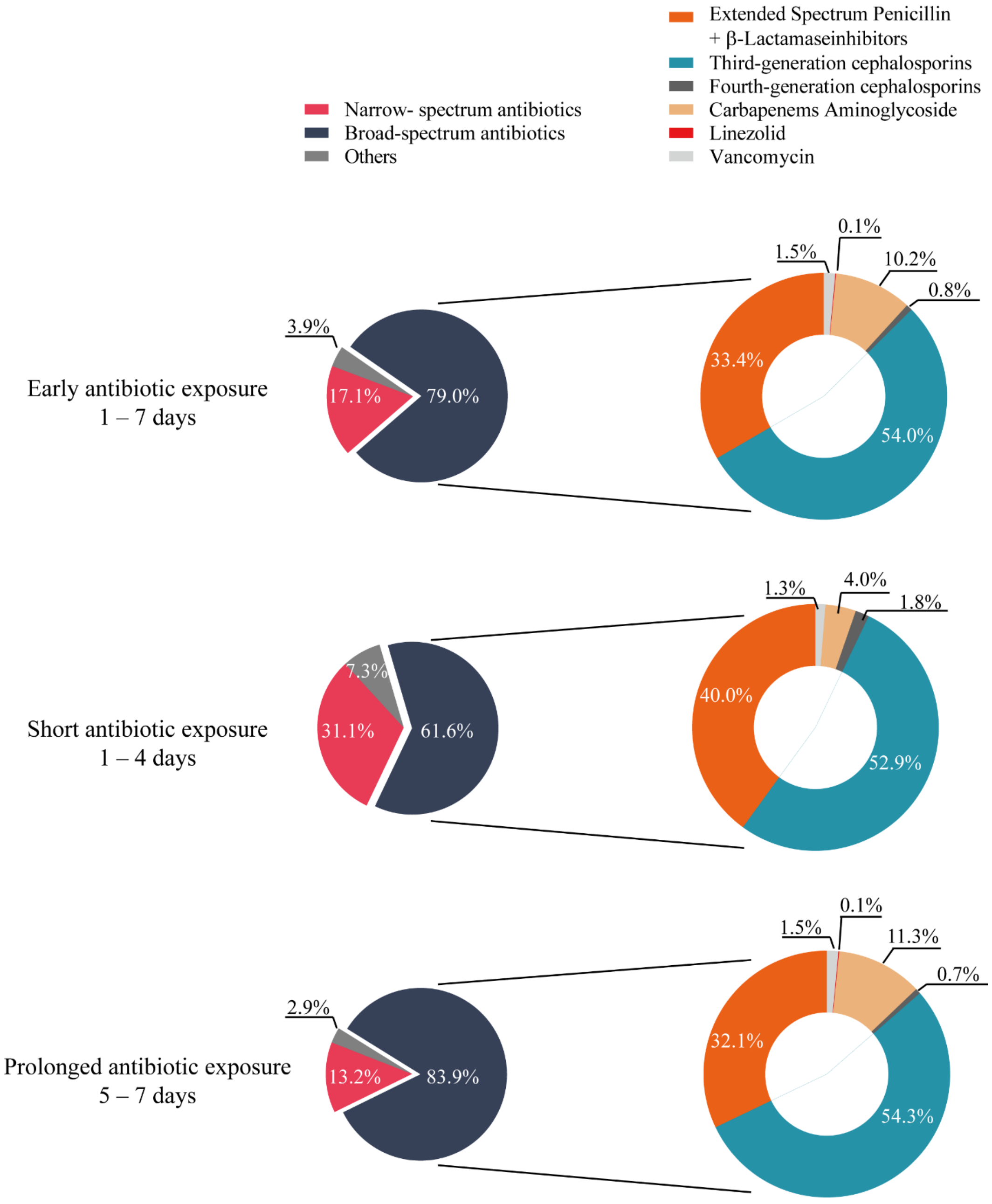

The utilization proportion of broad-spectrum antibiotics was significantly higher than that of narrow-spectrum antibiotics across different antibiotic exposure groups: early antibiotic exposure (1-7 days), short-term exposure (1-4 days) and prolonged exposure (5-7 days). Among broad-spectrum antibiotics, third-generation cephalosporins and extend spectrum penicillin with β-Lactamase inhibitors were most commonly administered.

© 2024 Shi W et al. *JAMA Network Open.*

eTable 2. Sensitivity Analysis after the Center Effect was Adjusted

**Outcomes**	**aOR (95% CI) Model 3**		
	**1–4 d VS None**	**5–7 d VS None**	**5–7 d VS 1-4 d**
Moderate to severe BPD or death	0.88 (0.67, 1.16)	1.23 (0.85, 1.78)	**1.40 (1.04, 1.89)**
Moderate to severe BPD in survivors at 36wk PMA	0.85 (0.63, 1.15)	1.19 (0.81, 1.76)	**1.40 (1.02, 1.92)**
Death prior to 36wk PMA	1.61 (0.82, 3.17)	1.73 (0.90, 3.34)	1.12 (0.72, 1.72)
Death before hospital discharge	1.28 (0.68, 2.40)	1.59 (0.83, 3.05)	1.24 (0.79, 1.95)

Abbreviations: PMA, postmenstrual age; BPD, bronchopulmonary dysplasia.

Generalized estimating equation models were used to adjusted for the center cluster effect. In model 3, GA, BW, SGA, sex, multiple pregnancy, maternal age, gestational diabetes, gestational hypertension or pre/eclampsia, antenatal corticosteroids use, magnesium sulfate use, Apgar score, intubation in delivery room, treatment with surfactant, NO, PDA requiring pharmacological treatment, mechanical ventilation treatment in 7 days after birth, and centers were adjusted. aOR (95% CI) was presented.

© 2024 Shi W et al. *JAMA Network Open.*

eFigure 3. Sensitivity Analysis According to the Different Classification Criteria

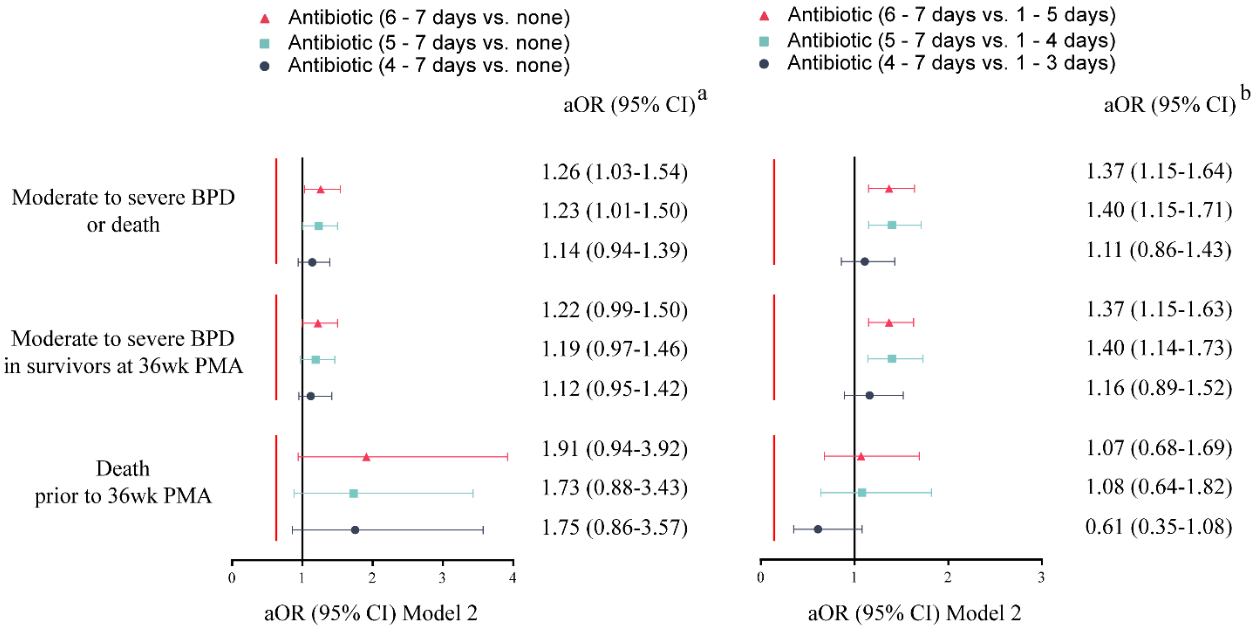

Abbreviations: PMA, postmenstrual age; BPD, bronchopulmonary dysplasia.

Three different cut-offs (3, 4, 5 calendar days) were used to define short and prolonged antibiotic exposure. Logistic regression model 2 was used to analyze the association between the prolonged antibiotic exposure and adverse outcomes. In model 2, GA, BW, SGA, sex, multiple pregnancy, maternal age, gestational diabetes, gestational hypertension or pre/eclampsia, antenatal corticosteroids use, magnesium sulfate use, Apgar score, intubation in delivery room, treatment with surfactant, NO, PDA requiring pharmacological treatment, and mechanical ventilation treatment in 7 days after birth were adjusted. aOR (95% CI) was presented.

[a] Reference is none antibiotic exposure.

[b] Reference is short-term antibiotic exposure.

© 2024 Shi W et al. *JAMA Network Open.*

eTable 3. Baseline Characteristics of Different Durations of Early Antibiotic Exposure Groups in the Propensity Score Matched Sample

Variables	Early Antibiotic Exposure		Absolute standardized mean difference	Early Antibiotic Exposure		Absolute standardized mean difference	Early Antibiotic Exposure		Absolute standardized mean difference
	None (N=833)	1-4days (N=833)		None (N=1168)	5-7days (N=1168)		1-4days (N=988)	5-7days (N=988)	
**Maternal characteristics, n(%)**									
Maternal age ≥35 y	205 (24.6)	173 (20.8)	0.093	280 (24.0)	357 (30.6)	0.151	213 (21.6)	207 (21.0)	0.015
Multiple pregnancy	262 (31.5)	260 (31.2)	0.005	372 (31.9)	352 (30.1)	0.038	320 (32.4)	295 (29.9)	0.054
Gestational diabetes	159 (19.1)	161 (19.3)	0.006	218 (18.7)	246 (21.1)	0.060	192 (19.4)	182 (18.4)	0.026
Antenatal corticosteroids	701 (84.2)	689 (82.7)	0.038	973 (83.3)	840 (72.0)	0.282	819 (82.9)	828 (83.8)	0.024
Gestational hypertension or Pre/eclampsia	406 (48.7)	406 (48.7)	0.000	618 (52.9)	628 (53.8)	0.017	446 (45.1)	440 (44.5)	0.012
Magnesium sulfate during delivery admission	510 (61.2)	514 (61.7)	0.010	723 (61.9)	638 (54.6)	0.147	605 (61.2)	590 (59.7)	0.031
**Neonatal characteristics, n(%)**									
Birth weight									
<1000g	69 (8.3)	84 (10.1)	0.059	83 (7.1)	149 (12.8)	0.159	103 (10.4)	87 (8.8)	0.053
>=1000g	764 (91.7)	749 (89.9)		1085 (92.9)	1046 (89.5)		885 (89.6)	901 (91.2)	
Gestational age, week									
<28	25 (3.0)	34 (4.1)	0.050	26 (2.2)	47 (4.0)	0.077	49 (5.0)	42 (4.3)	0.033
>=28	808 (97.0)	799 (95.9)		1142 (97.8)	1121 (96.0)		939 (95.0)	946 (95.7)	
SGA	273 (32.8)	272 (32.7)	0.003	483 (41.4)	453 (38.8)	0.057	289 (29.3)	275 (27.8)	0.031
Male sex	397 (47.7)	397 (47.7)	0.000	515 (44.1)	587 (50.3)	0.124	484 (49.0)	498 (50.4)	0.028
Apgar score <7 at 5 min	9 (1.1)	16 (1.9)	0.043	11 (0.9)	24 (2.1)	0.045	40 (4.1)	29 (2.9)	0.056
Intubation in delivery room	81 (9.7)	86 (10.3)	0.018	95 (8.1)	159 (13.6)	0.127	132 (13.4)	121 (12.3)	0.033
Mechanical ventilation treatment in 7 days after birth	109 (13.0)	130 (15.6)	0.067	119 (10.2)	204 (17.5)	0.151	170 (17.2)	169 (17.1)	0.003
Surfactant use	312 (37.5)	322 (38.7)	0.024	316 (27.1)	329 (28.2)	0.022	468 (47.4)	443 (44.8)	0.051
Nitric oxide use	1 (0.1)	0 (0.0)	0.022	1 (0.1)	2 (0.2)	0.011	3 (0.3)	3 (0.3)	0.000
PDA medication	97 (11.6)	101 (12.1)	0.015	127 (10.9)	171 (14.6)	0.108	118 (11.9)	104 (10.5)	0.044

© 2024 Shi W et al. *JAMA Network Open.*

PDA ligation	2 (0.2)	4 (0.5)	0.040	2 (0.2)	2 (0.2)	0.000	5 (0.5)	2 (0.2)	0.052

Abbreviations: SGA, Small-for-Gestational-Age; PDA, patent ductus arteriosus.

Three groups of infants were matched with each other with the closest propensity score on a 1:1 ratio by using the nearest neighbor greedy matching algorithm without replacement. The maximum distance between 2 matched infants (the caliper) was set to 0.2. Propensity score matching by GA, BW, SGA, sex, multiple pregnancy, maternal age, gestational diabetes, gestational hypertension or pre/eclampsia, antenatal corticosteroids use, magnesium sulfate use, Apgar score, intubation in delivery room, treatment with surfactant, NO, PDA requiring pharmacological treatment, and mechanical ventilation treatment in 7 days after birth.

© 2024 Shi W et al. *JAMA Network Open.*

eTable 4. Sensitivity Analysis of Different Durations of Early Antibiotic Exposure in the Propensity Score Matched Sample

Outcomes	Early Antibiotic Exposure, n (%)		aOR (95% CI); Model 2[b]	Early Antibiotic Exposure, n (%)		aOR (95% CI); Model 2[b]	Early Antibiotic Exposure, n (%)		aOR (95% CI); Model 2[b]
	**None (N=833)**	**1-4 days (N=833)**	**1–4 d VS None**	**None (N=1168)**	**5-7 days (N=1168)**	**5–7 d VS None**	**1-4 days (N=988)**	**5-7 days (N=988)**	**5–7 d VS 1-4 d**
Moderate to severe BPD or death	148 (17.8)	124 (14.9)	**0.70 (0.53, 0.94)**	172 (14.7)	265 (22.7)	**1.37 (1.09, 1.73)**	166 (16.8)	214 (21.7)	**1.52 (1.19, 1.94)**
Moderate to severe BPD in survivors at 36wk PMA	136 (16.7)	112 (13.6)	**0.70 (0.52, 0.95)**	160 (14.0)	234 (20.5)	**1.30 (1.03, 1.66)**	148 (15.3)	191 (19.8)	**1.51 (1.17, 1.94)**
Death prior to 36wk PMA	10 (1.2)	12 (1.4)	0.87 (0.35, 2.17)	10 (0.9)	29 (2.5)	**2.15 (1.02, 4.54)**	18 (1.8)	21 (2.1)	1.48 (0.74, 2.94)
Death before hospital discharge	14 (1.7)	13 (1.6)	0.65 (0.28, 1.50)	14 (1.2)	37 (3.2)	**1.99 (1.05, 3.78)**	19 (1.9)	26 (2.6)	1.72 (0.91, 3.27)

Abbreviations: PMA, postmenstrual age; BPD, bronchopulmonary dysplasia.

Propensity score model was used to analyze the association between the prolonged antibiotic exposure and adverse outcomes. aOR (95% CI) was presented.

© 2024 Shi W et al. *JAMA Network Open.*

eTable 5. Baseline Characteristics of Different Durations of Initial Antibiotic Therapy Groups (starting <72 hours of age) in the Propensity Score Matched Sample

Variables	Early Antibiotic Exposure		Absolute standardized mean difference	Early Antibiotic Exposure		Absolute standardized mean difference	Early Antibiotic Exposure		Absolute standardized mean difference
	None (N=686)	1-4days (N=686)		None (N=1168)	5-7days (N=1168)		1-4days (N=792)	5-7days (N=792)	
**Maternal characteristics, n(%)**									
Maternal age ≥35 y	160 (23.3)	145 (21.1)	0.053	280 (24.0)	312 (26.7)	0.063	176 (22.2)	169 (21.3)	0.021
Multiple pregnancy	223 (32.5)	237 (34.6)	0.043	372 (31.9)	372 (31.9)	0.000	274 (34.6)	261 (33.0)	0.034
Gestational diabetes	138 (20.1)	146 (21.3)	0.029	218 (18.7)	289 (24.7)	0.153	161 (20.3)	162 (20.5)	0.003
Antenatal corticosteroids	578 (84.3)	593 (86.4)	0.062	973 (83.3)	892 (76.4)	0.171	675 (85.2)	676 (85.4)	0.004
Gestational hypertension or Pre/eclampsia	327 (47.7)	316 (46.1)	0.032	618 (52.9)	565 (48.4)	0.091	340 (42.9)	327 (41.3)	0.033
Magnesium sulfate during delivery admission	421 (61.4)	443 (64.6)	0.066	723 (61.9)	613 (52.5)	0.190	494 (62.4)	488 (61.6)	0.016
**Neonatal characteristics, n(%)**									
Birth weight									
<1000g	50 (7.3)	60 (8.8)	0.049	83 (7.1)	131 (11.2)	0.118	77 (9.7)	77 (9.7)	0.038
>=1000g	636 (92.7)	629 (91.2)		1085 (92.9)	1037 (88.8)		715 (90.3)	724 (91.4)	
Gestational age, week									
<28	24 (3.5)	33 (4.8)	0.059	26 (2.2)	66 (5.7)	0.152	41 (5.2)	37 (4.7)	0.023
>=28	662 (96.5)	653 (95.2)		1142 (97.8)	1102 (94.3)		751 (94.8)	755 (95.3)	
SGA	208 (30.3)	198 (28.9)	0.033	483 (41.4)	454 (38.9)	0.056	209 (26.4)	205 (25.9)	0.011
Male sex	321 (46.8)	321 (46.8)	0.000	515 (44.1)	539 (46.2)	0.041	377 (47.6)	397 (50.1)	0.051
Apgar score <7 at 5 min	9 (1.3)	11 (1.6)	0.025	11 (0.9)	16 (1.4)	0.018	30 (3.8)	19 (2.4)	0.073
Intubation in delivery room	68 (9.9)	81 (11.8)	0.055	95 (8.1)	168 (14.4)	0.146	111 (14.0)	108 (13.6)	0.011
Mechanical ventilation treatment in 7 days after birth	86 (12.5)	104 (15.2)	0.071	119 (10.2)	183 (15.7)	0.114	128 (16.2)	125 (15.8)	0.010
Surfactant use	290 (42.3)	286 (41.7)	0.012	316 (27.1)	393 (33.7)	0.133	389 (49.1)	385 (48.6)	0.010
Nitric oxide use	1 (0.2)	2 (0.3)	0.024	1 (0.1)	1 (0.1)	0.000	3 (0.4)	3 (0.4)	0.000
PDA medication	73 (10.6)	79 (11.5)	0.028	127 (10.9)	170 (14.6)	0.106	87 (11.0)	75 (9.5)	0.048

© 2024 Shi W et al. *JAMA Network Open.*

PDA ligation	2 (0.3)	5 (0.7)	0.062	2 (0.2)	2 (0.2)	0.000	5 (0.6)	0 (0.0)	0.110

Abbreviations: SGA, Small-for-Gestational-Age; PDA, patent ductus arteriosus.

Three groups of infants were matched with each other with the closest propensity score on a 1:1 ratio by using the nearest neighbor greedy matching algorithm without replacement. The maximum distance between 2 matched infants (the caliper) was set to 0.2. Propensity score matching by GA, BW, SGA, sex, multiple pregnancy, maternal age, gestational diabetes, gestational hypertension or pre/eclampsia, antenatal corticosteroids use, magnesium sulfate use, Apgar score, intubation in delivery room, treatment with surfactant, NO, PDA requiring pharmacological treatment, and mechanical ventilation treatment in 7 days after birth.

© 2024 Shi W et al. *JAMA Network Open.*

eTable 6. Sensitivity Analysis of Different Durations of Initial Antibiotic Therapy (starting <72 hours of age) in the Propensity Score Matched Sample

Outcomes	Early Antibiotic Exposure, n (%)		aOR (95% CI); Model 2[b]	Early Antibiotic Exposure, n (%)		aOR (95% CI); Model 2[b]	Early Antibiotic Exposure, n (%)		aOR (95% CI); Model 2[b]
	None (N=686)	1-4 days (N=686)	1–4 d VS None	None (N=1168)	5-7 days (N=1168)	5–7 d VS None	1-4 days (N=792)	5-7 days (N=792)	5–7 d VS 1-4 d
Moderate to severe BPD or death	120 (17.5)	105 (15.3)	0.74 (0.54, 1.02)	172 (14.7)	245 (21.0)	**1.27 (1.00, 1.61)**	120 (15.2)	166 (21.0)	**1.70 (1.28, 2.26)**
Moderate to severe BPD in survivors at 36wk PMA	109 (16.3)	96 (14.2)	0.74 (0.53, 1.03)	160 (14.0)	217 (19.0)	1.18 (0.92, 1.51)	110 (14.1)	146 (18.8)	**1.61 (1.20, 2.16)**
Death prior to 36wk PMA	9 (1.3)	9 (1.3)	0.92 (0.35, 2.42)	10 (0.9)	26 (2.2)	**2.19 (1.03, 4.66)**	10 (1.3)	17 (2.2)	2.03 (0.88, 4.71)
Death before hospital discharge	13 (1.9)	9 (1.3)	0.61 (0.25, 1.51)	14 (1.2)	33 (2.8)	**1.95 (1.02, 3.72)**	10 (1.3)	23 (2.9)	**2.76 (1.25, 6.06)**

Abbreviations: PMA, postmenstrual age; BPD, bronchopulmonary dysplasia.

Propensity score model was used to analyze the association between the prolonged antibiotic exposure and adverse outcomes. aOR (95% CI) was presented.

© 2024 Shi W et al. *JAMA Network Open.*

eTable 7. Baseline Characteristics of Different Durations of Early Antibiotic Exposure in the Propensity Score Matched Sample with Imputation of Missing Data for Magnesium Sulfate During Delivery Admission

Variables	Early Antibiotic Exposure		Absolute standardized mean difference	Early Antibiotic Exposure		Absolute standardized mean difference	Early Antibiotic Exposure		Absolute standardized mean difference
	None (N=919)	1-4days (N=919)		None (N=1250)	5-7days (N=1250)		1-4days (N=1086)	5-7days (N=1086)	
**Maternal characteristics, n(%)**									
Maternal age ≥35 y	213 (23.2)	220 (23.9)	0.019	302 (24.2)	398 (31.8)	0.175	235 (21.6)	241 (22.2)	0.013
Multiple pregnancy	290 (31.6)	272 (29.6)	0.042	399 (31.9)	366 (29.3)	0.060	374 (32.0)	335 (30.9)	0.024
Gestational diabetes	183 (19.9)	168 (18.3)	0.042	231 (18.5)	263 (21.0)	0.065	204 (18.8)	189 (17.4)	0.035
Antenatal corticosteroids	757 (82.4)	732 (79.7)	0.069	1016 (81.3)	981 (78.5)	0.065	880 (81.0)	883 (81.3)	0.007
Gestational hypertension or Pre/eclampsia	435 (47.3)	411 (44.7)	0.053	660 (52.8)	636 (50.9)	0.038	483 (44.5)	473 (43.6)	0.019
Magnesium sulfate during delivery admission	509 (55.4)	500 (54.4)	0.020	723 (57.8)	629 (50.3)	0.150	605 (55.7)	582 (53.6)	0.043
**Neonatal characteristics, n(%)**									
Birth weight									
<1000g	77 (8.4)	90 (9.8)	0.047	91 (7.3)	181 (14.5)	0.206	112 (10.3)	81 (7.5)	0.094
>=1000g	842 (91.6)	829 (90.2)		1159 (92.7)	1069 (85.5)		974 (89.7)	1005 (92.5)	
Gestational age, week									
<28	28 (3.1)	33 (3.6)	0.025	30 (2.4)	63 (5.0)	0.117	51 (4.7)	35 (3.2)	0.069
>=28	891 (96.9)	886 (96.4)		1220 (97.6)	1187 (95.0)		1035 (95.3)	1051 (96.8)	
SGA	312 (34.0)	305 (33.2)	0.017	517 (41.4)	517 (41.4)	0.000	328 (30.2)	306 (28.2)	0.044
Male sex	442 (48.1)	442 (48.1)	0.000	564 (45.1)	588 (47.0)	0.039	531 (48.9)	531 (48.9)	0.000
Apgar score <7 at 5 min	12 (1.3)	17 (1.9)	0.027	15 (1.2)	41 (3.3)	0.082	46 (4.2)	36 (3.3)	0.046
Intubation in delivery room	91 (9.9)	113 (12.3)	0.01	109 (8.7)	183 (14.6)	0.137	144 (13.3)	144 (13.3)	0.000
Mechanical ventilation treatment in 7 days after birth	119 (13.0)	149 (16.2)	0.085	131 (10.5)	175 (14.0)	0.073	193 (17.8)	188 (17.3)	0.012
Surfactant use	328 (35.7)	347 (37.8)	0.041	333 (26.6)	389 (31.1)	0.090	504 (46.4)	511 (47.1)	0.013
Nitric oxide use	1 (0.1)	0 (0.0)	0.018	1 (0.1)	4 (0.3)	0.030	4 (0.4)	0 (0.0)	0.061
PDA medication	111 (12.8)	108 (11.8)	0.010	133 (10.6)	178 (14.2)	0.103	130 (12.0)	104 (9.6)	0.074

© 2024 Shi W et al. *JAMA Network Open.*

PDA ligation	2 (0.2)	4 (0.4)	0.038	3 (0.2)	2 (0.2)	0.018	5 (0.5)	1 (0.1)	0.071

Abbreviations: PMA, postmenstrual age; BPD, bronchopulmonary dysplasia.

Three groups of infants were matched with each other with the closest propensity score on a 1:1 ratio by using the nearest neighbor greedy matching algorithm without replacement. The maximum distance between 2 matched infants (the caliper) was set to 0.2. Propensity score matching by GA, BW, SGA, sex, multiple pregnancy, maternal age, gestational diabetes, gestational hypertension or pre/eclampsia, antenatal corticosteroids use, magnesium sulfate use, Apgar score, intubation in delivery room, treatment with surfactant, NO, PDA requiring pharmacological treatment, and mechanical ventilation treatment in 7 days after birth.

eTable 8. Sensitivity Analysis of Different Durations of Early Antibiotic Exposure in the Propensity Score Matched Sample with Imputation of Missing Data for Magnesium Sulfate During Delivery Admission

Outcomes	Early Antibiotic Exposure, n (%)		aOR (95% CI); Model 2[b]	Early Antibiotic Exposure, n (%)		aOR (95% CI); Model 2[b]	Early Antibiotic Exposure, n (%)		aOR (95% CI); Model 2[b]
	None (N=919)	1-4 days (N=919)	1–4 d VS None	None (N=1250)	5-7 days (N=1250)	5–7 d VS None	1-4 days (N=1086)	5-7 days (N=1086)	5–7 d VS 1-4 d
Moderate to severe BPD or death	156 (17.0)	146 (15.9)	0.83 (0.63, 1.09)	190 (15.2)	267 (21.4)	**1.29 (1.03, 1.61)**	188 (17.3)	234 (21.6)	**1.48 (1.18, 1.87)**
Moderate to severe BPD in survivors at 36wk PMA	143 (16.0)	132 (14.6)	0.82 (0.62, 1.09)	176 (14.5)	237 (19.4)	1.22 (0.97, 1.54)	169 (15.8)	212 (19.9)	**1.48 (1.17, 1.89)**
Death prior to 36wk PMA	10 (1.1)	14 (1.5)	1.18 (0.51, 2.76)	11 (0.9)	28 (2.2)	2.01 (0.97, 4.17)	19 (1.8)	21 (1.9)	1.37 (0.70, 2.70)
Death before hospital discharge	16 (1.7)	16 (1.7)	0.84 (0.40, 1.75)	17 (1.4)	36 (2.9)	1.66 (0.91, 3.05)	21 (1.9)	27 (2.5)	1.61 (0.86, 2.99)

Abbreviations: PMA, postmenstrual age; BPD, bronchopulmonary dysplasia.

Propensity score model was used to analyze the association between the prolonged antibiotic exposure and adverse outcomes. aOR (95% CI) was presented.

© 2024 Shi W et al. *JAMA Network Open.*

eTable 9. Baseline Characteristics of Participants in the Top 14 Centers that Implemented Relatively Stricter Criteria for Antibiotic Therapy

Variables	Early Antibiotic Exposure			*p*
	None (N=912)	1-4 days (N=511)	5-7 days (N=682)	
**Maternal characteristics, n/N (%)**				
Maternal age ≥35 y	229/908 (25.2)	104/509 (20.4)	186/676 (27.5)	0.019
Multiple pregnancy	308/912 (33.8)	158/511 (30.9)	198/682 (29.0)	0.124
Gestational diabetes	172/912 (18.9)	90/511 (17.6)	87/682 (12.8)	0.004
Antenatal corticosteroids	760/912 (83.3)	423/511 (82.8)	542/682 (79.5)	0.120
Gestational hypertension or Pre/eclampsia	483/912 (53.0)	249/511 (48.7)	353/682 (51.8)	0.306
Magnesium sulfate during delivery admission	566/874 (64.8)	325/481 (67.6)	428/632 (67.7)	0.398
**Neonatal characteristics, n/N (%)**				
Birth weight				
<1000g	77/912 (8.44)	67/511 (13.1)	137/682 (20.1)	<0.001
>=1000g	835/912 (91.6)	444/511 (86.9)	545/682 (79.9)	
Gestational age, week				
<28	30/912 (3.3)	39/511 (7.6)	60/682 (8.8)	<0.001
>=28	882/912 (96.7)	472/511 (92.4)	622/682 (91.2)	
SGA	333/912 (36.5)	139/511 (27.2)	163/682 (23.9)	<0.001
Male sex	430/912 (47.2)	247/511 (48.3)	396/682 (58.1)	<0.001
Apgar score <7 at 5 min	14/854 (1.6)	22/478 (4.6)	49/617 (7.9)	<0.001
Intubation in delivery room	116/912 (12.7)	82/511 (16.1)	171/682 (25.1)	<0.001
Mechanical ventilation treatment in 7 days after birth	133/912 (14.6)	108/511 (21.1)	280/682 (41.1)	<0.001
Surfactant use	271/912 (29.7)	245/511 (48.0)	394/682 (57.8)	<0.001
Nitric oxide use	1/912 (0.1)	3/511 (0.6)	3/682 (0.4)	0.204
PDA medication	128/912 (14.0)	81/511 (15.9)	108/682 (15.8)	0.517
PDA ligation	2/912 (0.2)	5/511 (1.0)	1/682 (0.2)	0.069

Abbreviations: SGA, Small-for-Gestational-Age; PDA, patent ductus arteriosus.

© 2024 Shi W et al. *JAMA Network Open.*

eTable 10. Sensitivity Analysis According to the Proportion of Infants with Prolonged Antibiotic Exposure

Outcomes	Center cluster 1		
	Early Antibiotic Exposure, n/N (%)		5-7 d VS None aOR (95% CI) in Model 2
	None（N=912）	5-7 d（N=682）	
Moderate to severe BPD or death	162/912 (17.8)	273/682 (40.0)	**1.66 (1.25, 2.22)**
Moderate to severe BPD in survivors at 36wk PMA	152/881 (17.3)	245/655 (37.4)	**1.56 (1.16, 2.10)**
Death prior to 36wk PMA	9/912 (1.0)	27/682 (4.0)	**2.78 (1.11, 6.95)**
Death before hospital discharge	13/912 (1.4)	30/682 (4.4)	**2.26 (1.01, 5.06)**

Abbreviations: PMA, postmenstrual age; BPD, bronchopulmonary dysplasia.

Center cluster 1: The top 14 centers that implemented relatively stricter criteria for antibiotic therapy.

Logistic regression model 2 was used to analyze the association between the prolonged antibiotic exposure and adverse outcomes. In model 2, GA, BW, SGA, sex, multiple pregnancy, maternal age, gestational diabetes, gestational hypertension or pre/eclampsia, antenatal corticosteroids use, magnesium sulfate use, Apgar score, intubation in delivery room, treatment with surfactant, NO, PDA requiring pharmacological treatment, and mechanical ventilation treatment in 7 days after birth were adjusted. aOR (95% CI) was presented.

© 2024 Shi W et al. *JAMA Network Open.*

eFigure 4. Stratified Analysis for Effects of Broad-spectrum or Narrow-spectrum Antibiotics Varied by the Duration of Antibiotics Exposure

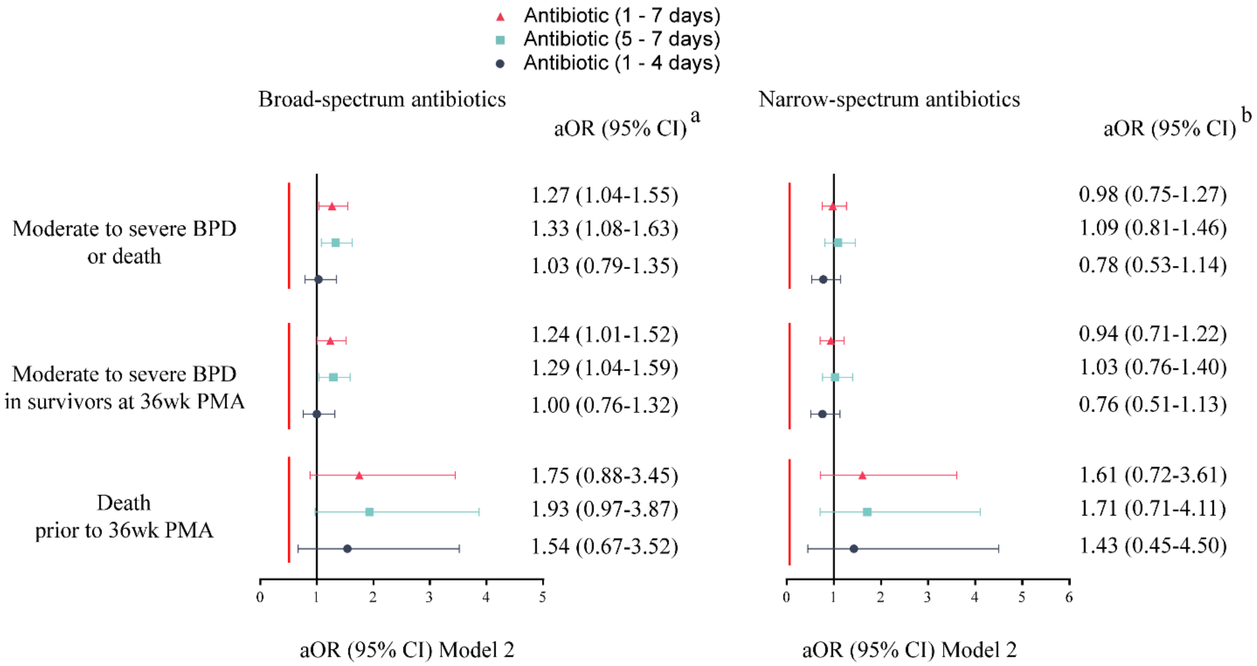

Abbreviations: PMA, postmenstrual age; BPD, bronchopulmonary dysplasia.

Logistic regression model 2 was used to analyze the association between the different durations of antibiotic exposure (1-4 days, 5-7 days, and 1-7 days) and adverse outcomes. Reference is none antibiotic exposure. In model 2, GA, BW, SGA, sex, multiple pregnancy, maternal age, gestational diabetes, gestational hypertension or pre/eclampsia, antenatal corticosteroids use, magnesium sulfate use, Apgar score, intubation in delivery room, treatment with surfactant, NO, PDA requiring pharmacological treatment, and mechanical ventilation treatment in 7 days after birth were adjusted. aOR (95% CI) was presented.

[a] Broad-spectrum antibiotic exposure group.

[b] Narrow-spectrum antibiotic exposure group.

© 2024 Shi W et al. *JAMA Network Open.*
