# Supplementary material for: Early Antibiotic Exposure and Bronchopulmonary Dysplasia in Very Preterm Infants at Low Risk of Early-Onset Sepsis
Source: JAMA Netw Open. 2024 Jun 27;7(6):e2418831. doi: 10.1001/jamanetworkopen.2024.18831 (PMC11211957; doi:10.1001/jamanetworkopen.2024.18831)
Supplement: Supplement 2. — Nonauthor Collaborators [file jamanetwopen-e2418831-s002.pdf]

Supplemental Online Content: Nonauthor Collaborators
*First name, last name, and suffix (if applicable) are required and will appear in PubMed.

*Group Name(s): Chinese Neonatal Network							
***First Name and Middle Initial(s)**	***Last Name**	***Suffix (eg, Jr, III)**	Academic Degrees	Institution	Location (city, state/province, country)	Role or Contribution, eg, chair, principal investigator	Group (if more than 1 Group listed in the byline) and/or Subgroup (eg, Steering Committee)
Chao	Chen		MD	Children's Hospital of Fudan University			
Yun	Cao		MD	Children's Hospital of Fudan University			
Xiuyong	Chen		MD	The Third Affiliated Hospital of Zhengzhou University			
Huyan	Zhang		MD	Guangzhou Women and Children's Medical Center			
Xiuying	Tian		MD	Tianjin Obstetrics & Gynecology Hospital			
Jingyun	Shi		MD	Gansu Provincial Maternity and Child Care Hospital			
Zhankui	Li		MD	Northwest Women's and Children's Hospital			
Chuanzhong	Yang		MD	Shenzhen Maternity and Child Health Care Hospital			
Ling	Liu		MD	Guizhou Women and Children's Hospital			
Zuming	Yang		MD	Suzhou Municipal Hospital affiliated to Nanjing Medical University			
Jianhua	Fu		MD	Shengjing Hospital of China Medical University			
Yong	Ji		MD	Children's Hospital of Shanxi			
Dongmei	Chen		MD	Quanzhou Women and Children's Hospital			
Changyi	Yang		MD	Fujian Women and Children's Medical Center			
Rui	Chen		MD	Children's Hospital of Nanjing Medical University			
Xiaoming	Peng		MD	Hunan Children's Hospital			
Ruobing	Shan		MD	Qingdao Women and Children's Hospital			
Shuping	Han		MD	Nanjing Maternity and Child Health Care Hospital			
Hui	Wu		MD	The First Bethune Hospital of Jilin University			
Lili	Wang		MD	The First Affiliated Hospital of Anhui Medical University			
Qiufen	Wei		MD	Women and Children's Hospital of Guangxi Zhuang Autonomous Region			
Mingxia	Li		MD	The First Affiliated Hospital of Xinjiang Medical University			
Yiheng	Dai		MD	Foshan Women and Children's Hospital			
Hong	Jiang		MD	The Affiliated Hospital of Qingdao University			
Wenqing	Kang		MD	Henan Children's Hospital			
Xiaohui	Gong		MD	Children's Hospital of Shanghai			
Xiaoyun	Zhong		MD	Chongqing Health Care Center for Women and Children			
Yuan	Shi		MD	Children's Hospital of Chongqing Medical University			

Supplemental Online Content: Nonauthor Collaborators
*First name, last name, and suffix (if applicable) are required and will appear in PubMed.

***First Name and Middle Initial(s)**	***Last Name**	***Suffix (eg, Jr, III)**	Academic Degrees	Institution	Location (city, state/province, country)	Role or Contribution, eg, chair, principal investigator	Group (if more than 1 Group listed in the byline) and/or Subgroup (eg, Steering Committee)
Shanyu	Jiang		MD	Wuxi Maternity and Child Healthcare Hospital			
Bing	Sun		MD	Children's Hospital of Soochow University			
Long	Li		MD	People's Hospital of Xinjiang Uygur Autonomous Region			
Zhenlang	Lin		MD	Yuying Children's Hospital Affiliated to Wenzhou Medical University			
Jiangqin	Liu		MD	Shanghai First Maternity and Infant Hospital			
Jiahua	Pan		MD	Anhui Provincial Hospital			
Hongping	Xia		MD	Xinhua Hospital affiliated to Shanghai Jiaotong University School of Medicine			
Xiaoying	Li		MD	Qilu Children's Hospital of Shandong University			
Falin	Xu		MD	The First Affiliated Hospital of Zhengzhou University			
Yinping	Qiu		MD	General Hospital of Ningxia Medical University			
Li	Ma		MD	Hebei Children's Hospital			
Ling	Yang		MD	Hainan Women and Children's Hospital			
Xiaori	He		MD	The second XiangYA hospital of Central South University			
Yanhong	Li		MD	Ningbo Women&Children Hospital			
Deyi	Zhuang		MD	Xiamen Children's Hospital			
Qin	Zhang		MD	Shaanxi Provincial People's Hospital			
Wenbin	Dong		MD	The Affiliated Hospital of Southwest Medical University			
Jianhua	Sun		MD	Shanghai Children's Medical Center affiliated to Shanghai Jiaotong University School of Medicine			
Kun	Liang		MD	First Affiliated Hospital of Kunming Medical University			
Huaiyan	Wang		MD	Changzhou Maternal and Children Health Care Hospital			
Jinxing	Feng		MD	Shenzhen Children's Hospital			
Liping	Chen		MD	Jiangxi Provincial Children's Hospital			
Xinzhu	Lin		MD	Xiamen Maternity and Child Health Care Hospital			
Chunming	Jiang		MD	Zhuhai Center for Maternal and Child Health Care			
Chuan	Nie		MD	Guangdong Women and Children's Hospital			
Linkong	Zeng		MD	Wuhan Chidren's Hospital			
Mingyan	Hei		MD	Beijing Children's Hospital of Capital Medical University			
Hongdan	Zhu		MD	Maternal and Children Hospital of Shaoxing			
Hongying	Mi		MD	The First People's Hospital of Yunnan Province			
Zhaoqing	Yin		MD	Dehong people's Hospital of Yunnan Province			
Hongxia	Song		MD	First Affiliated Hospital of Xian Jiaotong University			

Supplemental Online Content: Nonauthor Collaborators

*First name, last name, and suffix (if applicable) are required and will appear in PubMed.

***First Name and Middle Initial(s)**	***Last Name**	***Suffix (eg, Jr, III)**	Academic Degrees	Institution	Location (city, state/province, country)	Role or Contribution, eg, chair, principal investigator	Group (if more than 1 Group listed in the byline) and/or Subgroup (eg, Steering Committee)
Hongyun	Wang		MD	Inner Mongolia maternal and child health care hospital			
Dong	Li		MD	Dalian Municipal Women and Children’s Medical Center			
Yan	Gao		MD	Lianyungang Maternal and Children Health Hospital			
Yajuan	Wang		MD	Children's Hospital Affiliated to Capital Institute of Pediatrics			
Liying	Dai		MD	Anhui Children's Hoospital			
Liyan	Zhang		MD	Fuzhou Children’s Hospital of Fujian Province			
Yangfang	Li		MD	Kunming Children's Hospital			
Qianshen	Zhang		MD	Shenzhen Hospital of Hongkong University			
Guofang	Ding		MD	Peking Union Medical College Hospital			
Jimei	Wang		MD	Obstetrics & Gynecology Hospital of Fudan University			
Xiaoxia	Chen		MD	The Affiliated Hospital of Guizhou Medical University			
Zhen	Wang		MD	Qinghai Women and Children Hospital			
Zheng	Tang		MD	The International Peace Maternity & Child Health Hospital of China welfare institute			
Xiaomei	Zhang		MD	Inner Mongolia People's Hospital			
Xiaolan	Zhang		MD	Xiamen Humanity Hospital			
Fang	Wu		MD	Shanghai General Hospital			
Yanxiang	Chen		MD	The First People's Hospital of Yinchuan			
Ying	Wu		MD	The Third Hospital of Nanchang			
Joseph	Ting		MBBS	University of Alber			
