# Supplementary material for: Early Antibiotic Exposure and Bronchopulmonary Dysplasia in Very Preterm Infants at Low Risk of Early-Onset Sepsis
Source: JAMA Netw Open. 2024 Jun 27;7(6):e2418831. doi: 10.1001/jamanetworkopen.2024.18831 (PMC11211957; doi:10.1001/jamanetworkopen.2024.18831)
Supplement: Supplement 3. — Data Sharing Statement [file jamanetwopen-e2418831-s003.pdf]

Data Sharing Statement

Shi. Early Antibiotic Exposure and Bronchopulmonary Dysplasia in Very Preterm Infants at Low Risk of Early-Onset Sepsis. *JAMA Netw Open*. Published June 27, 2024.
doi:10.1001/jamanetworkopen.2024.18831

Data
Data available: No
